# Registered health problems and demographic profile of integrated textile factory workers in Ethiopia: a cross-sectional study

**DOI:** 10.1186/s12889-021-11556-4

**Published:** 2021-08-09

**Authors:** Yifokire Tefera Zele, Abera Kumie, Wakgari Deressa, Magne Bråtveit, Bente E. Moen

**Affiliations:** 1 Department of Preventive Medicine, School of Public Health, College of Health Sciences, Addis Ababa University, P O Box 9086, Addis Ababa, Ethiopia; 2 Department of Global Public Health and Primary Care, University of Bergen, Bergen, Norway; 3 Department of Global Public Health and Primary Care, Centre for International Health, Faculty of Medicine, University of Bergen, Årstadveien 21, 5009 Bergen, Norway

**Keywords:** Integrated textile, Respiratory disease, MSD, Injury, Clinical diagnosis, Work-related diseases, Ethiopia

## Abstract

**Background:**

Textile and garment factories are growing in low and middle-income countries as worldwide demand for inexpensive clothing increases each year. These integrated textile and garment production factories are often built-in areas with few workplaces and environmental regulations, and employees can be regularly exposed to workplace hazards with little regulatory oversight. Consequently, workers’ health may be significantly affected due to long term exposure to hazards. This study describes registered health problems and their association to work-related and personal factors among workers in integrated textile factories in Ethiopia.

**Methods:**

Institution-based cross-sectional study design was employed for this analysis. A one-year recording of worker’s clinical diagnoses (between March 2016 and February 2017) was gathered from the factory clinics of three integrated textile factories. Clinical diagnosis data was obtained as factory workers visited the clinics if feeling unwell. Sociodemographic characteristics and work-related information were obtained from the factory’s human resource departments. The sociodemographic and clinical diagnosis statuses of 7992 workers were analyzed. The association between the registered diagnoses and workplace factors (work in textile production, garment production and support process) and personal factors (age, sex and educational status) were studied using logistic regression analysis.

**Results:**

The average employee age and years of service were 40 years and 11 years respectively. 60% of workers were females, comprising of 4778 women. 66% of all workers (5276) had 27,320 clinical diagnoses. In total, this caused 16,993 absent working days due to sick leave. Respiratory diseases (34%) and musculoskeletal disorders (29%) were the most prevalent diagnoses, while bodily injuries were the cause of most work absences. Work department, sex and educational status are variables that were most significantly associated with higher prevalence of disease groups.

**Conclusions:**

About two-thirds of the integrated textile factory workers were diagnosed with different types of disease. The textile and garment production department workers were affected at a greater rate than the support process workers, indicating that some diseases may be related to workplace exposure. Further study should investigate rare chronic diseases such as cancer, heart diseases, renal diseases and diabetes.

## Background

The textile and garment sector is growing in low and middle-income countries (LMICs); currently, Sub-Saharan African countries seeking to industrialize are expanding textile production capabilities [1]. In Ethiopia, the integrated textile factories quoted as “Farm to Fashion” production [2], which comprise both textile production and garment processing have a higher priority in the textile and clothing value chain than stand-alone textile or garment factories. Integrated factories have comparative advantages of creating extensive employment opportunities, with some factories recruiting around 6000 workers per factory [2]. Furthermore, the departments are arranged in a chain to produce clothes from raw cotton; the fabrics produced at the weaving/knitting department use the yarn produced from the spinning department that processes raw cotton; then the final clothes made in the garment department use the fabrics produced from the finishing department [3]. The health risks present in the integrated processes may be different from separate stand-alone factories.

Traditionally, the textile industry has been known to cause respiratory diseases. Since the early 1900s, the main research regarding work and health in the textile and garment industry focused on the respiratory disease, byssinosis [4]. Several studies have indicated that processing raw cotton for clothing generates inhalable dust and endotoxins that might cause respiratory problems [5–7]. Respiratory issues are not the only hazard that textile and garment production employees are exposed to [8, 9]. Heavy machines and mechanical contact, manual labour, repetitive work, awkward working postures and increased pressure to produce can put workers at risk for work-related diseases, such as musculoskeletal disorders (MSDs) and traumatic injuries [10–12]. Some studies reported various health problems among workers in the textile and garment sector [13–16]. Nevertheless, several of these studies are reviews, and few of them are original researches that study health problems often assessed by the self-report.

As aforementioned, work in integrated textile factories may be associated with different health risks than those seen in factories with the sole purpose of fabric production [13, 17]. The textile department workers directly interact with raw cotton and dangerous machines to produce yarn and fabrics, implying an increased risk for respiratory diseases, injuries, and hearing impairment [9, 18–20]. Workers in the garment department process the fabrics; change the sizes, colours, and textures in various working conditions using different machines. This process increases the risk of MSDs attributive to repetitive work, unhealthy posture [10, 12] and cancer risk from the dyes [21]. The establishment of integrated textile factories has been rising in Ethiopia in the past years; yet there are no national statistics concerning occupational diseases and injuries to show the country’s workers’ health status.

Despite the prominent presence of health hazards in the textile industry, Occupational Health Services (OHS) is lacking in Ethiopia. According to ILO [22], only 5–10% of workers in developing countries have access to OHS. Nevertheless, several of the integrated textile factories in Ethiopia do have health clinics. These clinics are likely to diagnose work-related diseases but we have very little knowledge about their activities. Some factories have health clinics with registration books where information about workers’ health is available. The occupational disease study in the integrated textile factory helps to see the overall picture of health risks and associated impacts.

This study aims to describe the magnitude of registered health problems and the demographic profile of workers in the integrated textile factories from factory clinics during 1 year. Personal and workplace factors associated with the diseases were also identified and examined. Since workers visit the health clinics if they feel unwell or are injured, factory clinics can be a valuable source of information and knowledge about work-related diseases and injuries in the integrated textile factories of Low and Middle-Income Countries (LMICs). Using the ILO lists of occupational diseases [23], respiratory diseases, MSD, injuries and ear diseases/impaired hearing was the main focus of discussion in this paper.

## Methods

### Study design

A 1-year institution-based cross-sectional study was employed that collected workers’ health data from clinics in three integrated textile factories. In Ethiopia, integrated textile and garment factories aim to organize production by including the whole production line from raw cotton to clothing. The detailed production process in the integrated textile factories has been described in a previous study [19].

### Study settings

Three integrated textile factories participated in this study which fulfilled the inclusion criteria. One hundred thirty registered enterprises are active in the Ethiopian textile value chain; 20 grouped under integrated textile factories [24]. The selection criteria for the integrated textile factories for this study include the presence of four functional integrated production departments (spinning, weaving/knitting, finishing and garment), working in three shifts, the presence of health clinics for both emergency and non-emergency consultations and the availability of a health recording system for workers. Three integrated textile factories fulfilled the above inclusion criteria; Factory 1 and 2 are located in Amhara Regional State, both within 600 km from the capital Addis Ababa. Factory 3 is in Tigray Regional State 1300 km North of Addis Ababa. These three factories 1, 2 and 3, were established in 1961, 1986 and 1992, respectively and had clinics from inception.

Each clinic, located within the factory compound provides similar services, including an outpatient department, emergency admission, laboratory facility and drug dispensary. Factory 3 clinic has diagnosed more workers than factory 1 and factory 2 clinics due to the high number of workers employed in factory 3. Each clinic has a general physician, 2–3 health officers, 5–7 nurses, 2–4 health assistants, 2–3 laboratory technicians, and 1–3 pharmacists. The clinics are primary healthcare centres; hence they have a referral system to hospitals and specialized clinics for advanced diagnosis and treatment. Since the factories function in three shifts, the clinics are open 24 h for consultations, but emergency service is available only for night shift workers. Overall, non-emergency cases can visit the clinic in the daytime outside of normal working hours; however, workers can visit the clinic for emergency health conditions while at work with approval from a supervisor.

### Study population

**Fig. 1:**
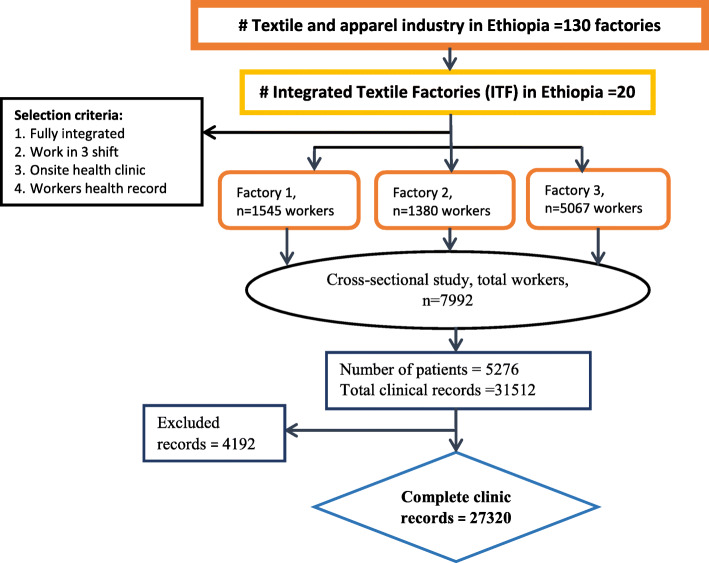
Illustrative diagram of the study population and data collection procedure of the integrated textile factories

The population study comprised all workers in the three integrated textile factories; these are workers in factories 1, 2 and 3 with 1545, 1380, and 5067 employees respectively (Fig. 1). Workers were broadly categorized into three groups: textile production department, garment production department and support department. The textile department included workers in spinning, weaving/knitting and finishing; the garment department included workers in garment production, and the support department included workers from administrative and maintenance services. The main work activities in the textile, garment and support are yarn/fabric making, cloth production and technical/administrative roles, respectively. Therefore, workers in the various department may have different exposure risks. Workers in the textile department are exposed to dust, noise, and dangerous machines, those in the garment department workers are predominately exposed to ergonomic hazards and chemicals, whereas the support department workers are exposed to mechanical and office related hazards. Furthermore, support department workers occasionally enter the production department for machine or production-related support issues that may expose them to hazards in the production sections; otherwise, they work regularly in the office or their workshops.

### Data collection

Data was collected from the clinics and human resource offices of the factories. Each worker’s personal and working department profile is available from the human resource registration in a Microsoft Excel database that contains a list with a unique identification number, date of birth, sex, education, working department, and number of years employed.

Workers’ Medical information was obtained from the factory clinics. Each employee has a patient card labelled with their name and a unique identification number, which is the same as the one used in the human resource database. The registration on the patient card includes information about the date of consultation, type of diagnoses and number of sick leave days absent for each worker. During sick leave, a worker may be admitted to the clinic or given medication to administer at home with paid regular wage. Depending on the medical procedure, the worker may also share the medical cost. All clinical consultations of workers from 1st March 2016 to 28th February 2017 were extracted from the health archives of the factory clinics and registered manually in a logbook prepared for this research purpose.

In this study, no specific diagnostic code system was used. The clinic physicians used many diagnosis types, so the diagnoses were grouped into the following comprehensive categories: respiratory diseases, injuries, musculoskeletal disorders, allergy, ear diseases, eye diseases, gastrointestinal infections, mouth diseases, peptic ulcer diseases, reproductive health problems, skin diseases, neurologic and psychiatric diseases, and other health conditions. These categories have various groups of health conditions and were described as follows. Respiratory diseases included bronchitis, asthma, pneumonia, pulmonary tuberculosis, and upper respiratory tract infections. Injuries included fractures, cuts, dislocations, burns, swellings, soft tissue injuries, and lacerations. Musculoskeletal disorders included back pain, neck stiffness, disc prolapse, joint pain, leg pain, myalgia and arthritis. Allergies included allergic rhinitis, allergic conjunctivitis, allergic sinusitis, allergic reaction, skin allergy and food allergy. Ear diseases included otitis media, ear infection, vertigo, ear pain and hearing problems. Eye diseases included conjunctivitis, trachoma, chalazion, presbyopia, pterygium, blepharitis, blurred vision, short sight, glaucoma. Gastrointestinal infections included intestinal parasites and dysentery. Mouth diseases included dental caries, tonsillitis, oral candidiasis, tooth bleeding, glossitis and gum infection. Peptic ulcer diseases included gastritis, epigastric burn, hernia, dyspepsia and gastrointestinal disorder. Reproductive health problems included mastitis, pelvic inflammatory disease, dysfunctional uterine bleeding, abortion, genital ulcer, cyst, breast tumour, fistula, scrotal swelling, dysmenorrhea, cervical cancer, sexually transmitted infection and vaginal bleeding. Skin diseases included dermatitis, herpes zoster, herpes simplex, wart, skin rash, scabies, cellulitis, tinea corporis, boils, melasma, contact dermatitis and tinea capitis. Neurologic and psychiatric diseases included migraine, neuralgia, nerve problems, peripheral neuropathy, sciatica, anxiety, depression, mental disturbance, and psychosis. Other diseases included cancer, cardiac, kidney, goitre, chronic liver disease, chronic osteomyelitis, rectal prolapse, appendicitis, tumour, bone problem and insomnia—some disease-specific diagnoses used for diabetics, hypertension, anemia, hemorrhoids, and urinary tract infection.

Clinic consultation for antenatal services, chronic disease follow-ups and visits to change the treatment regime were excluded from the study. From 31,512 clinic consultations, 4192 were excluded due to incomplete information (Fig. 1). A worker may visit the clinic for a new diagnosis or a previously known health problem requiring medical treatment. A worker may visit the clinic for consultations more than once for the same diagnosis category at a different time or for a different diagnosis. Thus, a worker’s clinical investigation of a new disease or further diagnosis for medical treatment to a previously known health condition within 1 year is counted. Four nurses based in each clinic (12 nurses) participated in the data collection with 2 days of training for this work by the principal investigator. The data extraction is checked for consistency and completeness by the first author, and two clerks entered the clinical diagnosis data from the logbook into the Microsoft Excel spreadsheet.

### Measurement of variables

During analysis, the diagnostic categories are the outcome/dependent variables, while workplace and personal factors are the independent variables. The variable work department is one of the workplace factors categorized into three groups; textile, garment and support. The work department (textile production, garment production and support process) that represents and describes the peculiar nature of work-related circumstances found in each department, the machines, raw materials, work process, product, and the physical and psychosocial work environment. The textile and garment department workers are directly engaged in the production, and are more exposed to work environment hazards than the support department; hence the support department is a reference group during analysis. Similarly, the personal factors, education is grouped into three categories: able to read and write, completed grade 1-10th, and those at the college level and above. Further, age is treated as a continuous variable, while male, higher education group and factory 3 are reference categories (Table 3).

### Statistical analysis

Clinical and demographic data sources were merged in an Excel database, then transferred to SPSS version 22 (SPSS, Chicago, IL, USA) for cleaning and analysis.

Two unique identification variables were created in the database; person specific unique identification and disease specific unique identification. The person specific unique identification was given for each study participant, and can be repeated in the database if the person has been diagnosed more than one time. Accordingly, 7992-person specific unique identifications with 27,320 records were produced. This unique identification helped to analyze and describe the independent variables of personal and workplace factors. Disease specific unique identification variables were also produced by combining the person specific unique identification variable with the specific disease code. This helped to calculate the specific disease prevalence and the number of repeated diagnosis for a specific disease.

**Fig. 2:**
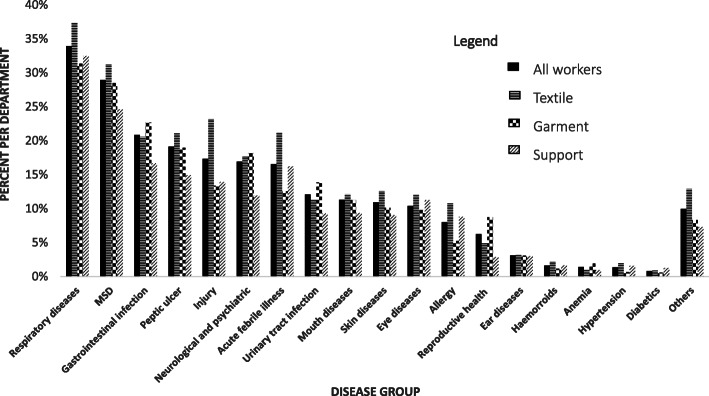
Prevalence of disease groups by working department in the integrated textile factories of Ethiopia, 2016–2017

Descriptive statistics were used, with an arithmetic mean utilized for the continuous variables such as age, years of service at work and number of days absent. Frequency and percentage were also used to describe categorical variables such as sex, education, health status and work force in each factory. Each of the independent variable mentioned above was stratified and presented by the three working departments (Textile, Garment and Support) (Table 1). Also, the percentage of each dependent variable, the 18 disease groups, was stratified by working department and presented in bar graph (Fig. 2). Furthermore, the prevalence, number and of repeated diagnosis, and number of sickness absence days for each disease outcome were calculated and presented in Table 2.

**Table 1:** Distribution of demographic and diagnostic characteristics of workers by working department in the integrated textile factories 2016–2017

Variable	Category	Working Department			Overall
		Textile	Garment	Support	
		AM, SD (Range)	AM, SD (Range)	AM, SD (Range)	AM, SD (Range)
**Age,** Years		40, 12 (18–69)	38, 8 (21–67)	44, 12 (21–69)	40, 10 (18–69)
**Service year,** Years		13, 12 (1–44)	9, 8 (1–46)	13, 11 (1–44)	11, 10 (1–46)
		**n (%)**	**n (%)**	**n (%)**	**n (%)**
**Sex**	Male	1847 (58)	461 (13)	919 (71)	3214 (40)
	Female	1322 (42)	3060 (87)	383 (29)	4778 (60)
**Education**	Read and Write	246 (8)	171 (5)	138 (11)	555 (7)
	Grade 10 and below	1344 (42)	2436 (69)	406 (31)	4188 (52)
	College and above	1579 (50)	914 (26)	758 (58)	3249 (41)
**Worker health status**	Diagnosed	2224 (70)	2284 (65)	768 (59)	5276 (66)
	Not diagnosed	945 (30)	1237 (35)	534 (41)	2716 (34)
**Factory**	Factory #1	930 (29)	344 (10)	271 (21)	1545 (19)
	Factory #2	778 (25)	326 (9)	276 (21)	1380 (17)
	Factory #3	1461 (46)	2851 (81)	755 (58)	5067 (63)

*AM* Arithmetic mean, *SD* Standard deviation

**Table 2:** Prevalence of diseases, frequency of consultations and sickness absence among all workers (*n* = 7992) in 2016–2017

Disease group^**a**^	Prevalence of disease among the workers (n_**1**_ = 7992 workers)		Number of diagnosis (n_**2**_ = 27,320)		Sick leave^**d**^ (workdays absence)
	Cases^**b**^	%	Diagnosis count^**c**^	%	
Respiratory diseases	2711	34	4691	17.2	2327
Musculoskeletal diseases	2312	29	3916	14.3	1949
Gastrointestinal infection	1666	21	2294	8.4	931
Peptic ulcer	1529	19	2306	8.4	1323
Injury	1388	17	1918	7.0	2951
Acute febrile illness	1325	17	2210	8.1	1822
Neurological and Psychiatric	1335	17	1894	6.9	810
Urinary tract infection	968	12	1358	5.0	527
Skin diseases	876	11	1140	4.2	475
Mouth disease	905	11	1129	4.1	397
Eye diseases	833	10	1049	3.8	482
Allergy	642	8	806	3.0	416
Reproductive health problem	502	6	679	2.5	547
Ear problem	251	3	298	1.1	92
Hemorrhoids	132	2	172	0.6	77
Hypertension	111	1	131	0.5	200
Anemia	115	1	127	0.5	59
Diabetic	67	1	105	0.4	99
Others	798	10	1097	4.0	1509

^a^One worker may be diagnosed for more than one disease group

^b^Number of workers diagnosed with the disease at least one time

^c^Number of diagnosis by the disease group, a worker may be diagnosed for more than one time for the disease group

^d^Number of workdays absence due to sick leave for each disease group

A disease diagnosis at least one time per worker was used in the logistic regression analysis. Univariate logistic regression analysis was performed for each disease outcome with an independent variable to determine whether to include the variable in the model. The selection of the variable is based on a *P*-value < 0.2. A multicollinearity diagnostic test was performed among the independent variables; worker’s age and service years were significantly correlated (*r* = 0.8, *p* <  0.001); consequently, we decided not to add the variable service years in the logistic regression analyses. Finally, the multivariable binary logistic regressions analysis was employed to identify any of the workplace and personal factors which had significant association with each disease outcome based on AOR with 95% CI and *P*-value < 0.05. This logistic regression analysis procedure was repeated for each disease outcome in Table 3.

**Table 3:** Multivariate analysis results of disease group among workers in the integrated textile factories (*n* = 7992)

Disease	Factor variables	Bivariate analysis COR (95% CI)	Multivariate analysis AOR (95% CI)	***P***-value
Respiratory diseases	Textile	1.24 (1.08–1.42)	1.22 (1.06–1.41)^*^	0.007
	Garment	0.95 (0.83–1.09)	1.30 (1.11–1.52)^*^	0.001
	Female	0.91 (0.82–1.00)	1.13 (1.01–1.26)^*^	0.033
	Read and write	1.48 (1.25–1.77)	1.05 (0.87–1.27)	0.619
	Factory 1	2.32 (2.06–2.61)	2.72 (2.39–3.10)^*^	< 0.001
	Factory 2	2.92 (2.58–3.30)	2.29 (1.98–2.65)^*^	< 0.001
	Age	1.03 (1.02–1.03)	1.02 (1.02–1.03)^*^	< 0.001
Musculoskeletal disorders	Textile	1.39 (1.20–1.61)	1.41 (1.21–1.65)^*^	< 0.001
	Garment	1.22 (1.05–1.41)	1.67 (1.41–1.98)^*^	< 0.001
	Female	1.11 (1.01–1.23)	1.37 (1.21–1.54)^*^	< 0.001
	Read and write	2.14 (1.79–2.54)	1.52 (1.25–1.84)^*^	< 0.001
	Factory 1	2.12 (1.88–2.40)	2.88 (2.52–3.30)^*^	< 0.001
	Factory 2	2.89 (2.55–3.28)	2.08 (1.78–2.42)^*^	< 0.001
	Age	1.03 (1.03–1.04)	1.03 (1.03–1.04)^*^	< 0.001
Injuries	Textile	1.86 (1.56–2.22)	1.79 (1.49–2.16)^*^	< 0.001
	Garment	0.96 (0.80–1.15)	1.66 (1.35–2.05)^*^	< 0.001
	Female	0.63 (0.56–0.70)	0.82 (0.71–0.94)^*^	0.004
	Read and write	1.62 (1.32–1.98)	1.38 (1.10–1.73)^*^	0.005
	Factory 1	4.81 (4.18–5.54)	4.90 (4.20–5.70)^*^	< 0.001
	Factory 2	3.39 (2.92–3.95)	2.60 (2.17–3.11)^*^	< 0.001
	Age	1.01 (1.01–1.02)	1.01 (1.01–1.02)^*^	< 0.001
Gastrointestinal	Textile	1.29 (1.09–1.53)	1.29 (1.08–1.53)^*^	0.004
	Garment	1.47 (1.24–1.73)	1.67 (1.39–2.01)^*^	< 0.001
	Female	1.15 (1.03–1.29)	1.11 (0.98–1.26)	0.110
	Read and write	1.25 (1.02–1.53)	1.10 (0.89–1.36)	0.388
	Factory 1	1.31 (1.14–1.50)	1.57 (1.35–1.82)^*^	< 0.001
	Factory 2	1.48 (1.28–1.70)	1.39 (1.18–1.65)^*^	< 0.001
	Age	1.01 (1.01–1.02)	1.01 (1.01–1.02)^*^	< 0.001
Peptic ulcer	Textile	1.52 (1.28–1.81)	1.36 (1.14–1.64)^*^	0.001
	Garment	1.33 (1.12–1.59)	1.56 (1.28–1.90)^*^	< 0.001
	Female	1.39 (1.24–1.56)	1.79 (1.56–2.05)^*^	< 0.001
	Read and write	1.66 (1.37–2.02)	1.19 (0.96–1.48)	0.12
	Factory 1	2.55 (2.22–2.93)	3.45 (20.96–4.02)^*^	< 0.001
	Factory 2	3.12 (2.71–3.59)	2.84 (2.39–3.38)^*^	< 0.001
	Age	1.02 (1.02–1.03)	1.02 (1.01–1.03)^*^	< 0.001
Acute Febrile Illness	Textile	1.38 (1.17–1.64)	1.15 (0.95–1.38)	0.146
	Garment	0.74 (0.62–0.89)	1.19 (0.97–1.46)	0.100
	Female	0.81 (0.72–0.91)	1.29 (1.12–1.48)^*^	< 0.001
	Read and write	1.76 (1.44–2.15)	1.42 (1.13–1.78)^*^	0.003
	Factory 1	8.28 (7.12–9.62)	9.67 (8.19–11.40)^*^	< 0.001
	Factory 2	5.66 (4.83–6.64)	4.82 (3.99–5.83)^*^	< 0.001
	Age	1.02 (1.01–1.02)	1.01 (1.01–1.02)^*^	< 0.001
Allergies	Textile	1.25 (1.00–1.55)	1.16 (0.92–1.47)	0.206
	Garment	0.57 (0.45–0.73)	1.09 (0.83–1.43)	0.527
	Female	0.61 (0.52–0.72)	0.94 (0.78–1.12)	0.468
	Read and write	2.16 (1.69–2.77)	1.07 (0.81–1.41)	0.621
	Factory 1	4.54 (3.62–5.69)	4.76 (3.74–6.06)^*^	< 0.001
	Factory 2	10.20 (8.28–12.55)	7.51 (5.88–9.59)^*^	< 0.001
	Age	1.05 (1.05–1.06)	1.02 (1.01–1.03)^*^	< 0.001
Mouth diseases	Textile	1.34 (1.08–1.66)	1.26 (1.01–1.57)^*^	0.041
	Garment	1.23 (1.00–1.52)	1.19 (0.94–1.51)	0.150
	Female	1.35 (1.16–1.56)	1.54 (1.30–1.81)^*^	< 0.001
	Read and write	1.22 (0.95–1.58)	1.02 (0.78–1.34)	0.877
	Factory 1	1.44 (1.21–1.71)	1.65 (1.37–1.99)^*^	< 0.001
	Factory 2	1.67 (1.40–1.98)	1.55 (1.25–1.91)^*^	< 0.001
	Age	1.01 (1.01–1.02)	1.01 (1.00–1.02)^*^	0.005
Anemia	Textile	1.09 (0.57–2.06)	0.99 (0.52–1.90)	0.976
	Garment	1.97 (1.09–3.58)	1.17 (0.62–2.22)	0.631
	Female	3.23 (1.99–5.25)	2.83 (1.67–4.79)^*^	< 0.001
	Read and write	1.43 (0.76–2.67)	@	
	Factory 1	0.08 (0.02–0.31)	0.10 (0.02–0.41)^*^	0.001
	Factory 2	1.21 (0.79–1.87)	1.59 (0.99–2.55)	0.053
	Age	1.00 (0.99–1.02)	@	
Hypertension	Textile	1.31 (0.80–2.15)	1.28 (0.77–2.14)	0.338
	Garment	0.42 (0.23–0.76)	1.21 (0.65–2.24)	0.549
	Female	1.11 (0.75–1.63)	@	
	Read and write	3.41 (2.12–5.48)	1.23 (0.74–2.05)	0.426
	Factory 1	36.84 (11.28–120.29)	42.48 (12.90–139.94)^*^	< 0.001
	Factory 2	97.01 (30.55–308.10)	38.29 (11.59–126.49)^*^	< 0.001
	Age	1.11 (1.09–1.13)	1.07 (1.05–1.09)^*^	< 0.001
Skin disease	Textile	1.45 (1.17–1.80)	1.43 (1.15–1.78)^*^	0.001
	Garment	1.13 (0.91–1.41)	1.26 (0.99–1.60)	0.061
	Female	0.86 (0.74–0.99)	0.89 (0.76–1.05)	0.165
	Read and write	1.00 (0.76–1.32)	@	
	Factory 1	1.40 (1.18–1.66)	1.31 (1.10–1.57)^*^	0.003
	Factory 2	1.09 (0.90–1.32)	1.04 (0.85–1.26)	0.728
	Age	1.00 (1.00–1.01)	@	
Urinary tract infection	Textile	1.25 (1.00–1.55)	1.04 (0.82–1.31)	0.732
	Garment	1.57 (1.28–1.94)	1.24 (0.98–1.57)	0.076
	Female	3.64 (3.06–4.33)	5.09 (4.19–6.17)^*^	< 0.001
	Read and write	2.09 (1.69–2.60)	1.36 (1.06–1.73)^*^	0.015
	Factory 1	1.30 (1.09–1.55)	2.29 (1.88–2.79)^*^	< 0.001
	Factory 2	2.41 (2.06–2.83)	1.94 (1.57–2.41)^*^	< 0.001
	Age	1.04 (1.03–1.04)	1.05 (1.04–1.05)^*^	< 0.001
Ear diseases	Textile	1.06 (0.73–1.54)	@	
	Garment	1.02 (0.71–1.47)	@	
	Female	0.80 (0.62–1.03)	1.02 (0.78–1.33)	0.872
	Read and write	0.67 (0.37–1.20)	0.60 (0.33–1.10)	0.101
	Factory 1	2.62 (1.98–3.47)	2.85 (2.13–3.81)^*^	< 0.001
	Factory 2	1.39 (0.98–1.98)	1.05 (0.70–1.56)	0.817
	Age	1.01 (1.00–1.03)	1.03 (1.01–1.04)^*^	< 0.001
Eye diseases	Textile	1.09 (0.89–1.33)	1.24 (1.01–1.53)^*^	0.04
	Garment	0.74 (0.60–0.91)	0.94 (0.74–1.19)	0.587
	Female	0.69 (0.60–0.80)	0.80 (0.67–0.94)^*^	0.008
	Read and write	1.29 (0.99–1.67)	1.21 (0.92–1.60)	0.169
	Factory 1	1.18 (0.99–1.41)	1.12 (0.93–1.35)	0.246
	Factory 2	0.89 (0.73–1.09)	0.58 (0.46–0.73)^*^	< 0.001
	Age	1.01 (1.01–1.02)	1.02 (1.01–1.03)^*^	< 0.001
Haemorroids	Textile	1.31 (0.81–2.12)	1.42 (0.87–2.34)	0.163
	Garment	0.69 (0.41–1.17)	1.07 (0.60–1.91)	0.823
	Female	0.59 (0.42–0.83)	0.75 (0.51–1.11)	0.149
	Read and write	1.35 (0.74–2.45)	@	
	Factory 1	1.82 (1.19–2.77)	1.70 (1.09–2.66)^*^	0.020
	Factory 2	2.16 (1.43–3.27)	1.38 (0.84–2.26)	0.198
	Age	1.03 (1.01–1.04)	1.02 (1.01–1.04)^*^	0.012
Reproductive health problems	Textile	1.74 (1.21–2.49)	1.33 (0.92–1.93)	0.127
	Garment	3.22 (2.28–4.54)	1.27 (0.89–1.82)	0.194
	Female	14.76 (9.77–22.30)	14.64 (9.58–22.39)^*^	< 0.001
	Read and write	1.00 (0.70–1.43)	@	
	Factory 1	0.87 (0.68–1.11)	@	
	Factory 2	1.02 (0.80–1.30)	@	
	Age	0.99 (0.98–1.00)	1.01 (1.00–1.02)	0.219
Neurologic and psychiatric diseases	Textile	0.97 (0.65–1.45)	0.95 (0.64–1.43)	0.819
	Garment	1.37 (0.94–2.00)	1.00 (0.66–1.51)	0.986
	Female	1.73 (1.30–2.29)	1.60 (1.16–2.21)^*^	0.004
	Read and write	0.94 (0.56–1.57)	@	
	Factory 1	0.53 (0.36–0.79)	0.60 (0.40–0.90)^*^	0.015
	Factory 2	0.81 (0.57–1.15)	0.90 (0.62–1.31)	0.590
	Age	1.00 (0.99–1.01)	@	
Diabetics	Textile	0.70 (0.39–1.29)	0.76 (0.41–1.43)	0.398
	Garment	0.46 (0.24–0.87)	1.25 (0.61–2.57)	0.549
	Female	0.61 (0.38–0.99)	1.22 (0.71–2.11)	0.466
	Read and write	1.32 (0.57–3.07)	@	
	Factory 1	24.15 (10.26–56.84)	33.49 (13.94–80.49)^*^	< 0.001
	Factory 2	11.15 (4.42–28.14)	5.06 (1.89–13.55)^*^	0.001
	Age	1.07 (1.04–1.09)	1.08 (1.05–1.10)^*^	< 0.001
Overall morbidity	Textile	1.64 (1.43–1.87)	1.66 (1.44–1.92)^*^	< 0.001
	Garment	1.28 (1.13–1.46)	1.65 (1.42–1.93)^*^	< 0.001
	Female	1.08 (0.98–1.18)	1.33 (1.18–149)^*^	< 0.001
	Read and write	1.78 (1.45–2.18)	1.42 (1.14–1.76)^*^	0.002
	Factory 1	2.58 (2.26–2.95)	3.32 (2.87–3.84)^*^	< 0.001
	Factory 2	2.56 (2.22–2.94)	1.86 (1.59–2.19)^*^	< 0.001
	Age	1.02 (1.02–1.03)	1.03 (1.02–1.04)^*^	< 0.001

*COR* Crude odds ratio, *AOR* Adjusted odds ratio, *CI* Confidence interval

^@^ Variables with *P* > 0.2 in bivariate not included in multivariate; * Significance level at *P* < 0.05; Support department, male, higher educational status and factory 3 are reference group

## Results

### Demographic and work characteristics

A total of 7992 workers were included in the analysis. The average and (standard deviation) age of workers was 40 [11] years with an age range of 18–69 years. The proportion of workers in the textile, garment and support departments was 40, 44 and 16%, respectively. 60% of the workers were females; the proportions of female workers being 87, 40, and 33% among the garment, textile, and support departments respectively. 52% of workers had completed education level grade 1–10, while 7 % of workers had no formal education but could read and write. The highest proportion of uneducated workers was among the support department (13%) and lowest among the garment department (5%). About (66%) of workers had at least one diagnosis during the 1 year of the observation period (Table 1).

### Prevalence of registered diseases

A total of 27,320 consultations took place with a total of 5276 diagnosed workers, equivalent to five consultations per worker during the study period. The highest proportion of total consultations was due to respiratory diseases (17%). Further, the highest prevalence of diseases diagnosed at least one time per individual worker were respiratory diseases (34%), followed by MSD (29%), gastrointestinal infection (21%), peptic ulcer disease (19%) and injury (17%) (Table 2).

A total of 16,993 workdays were registered as sickness absence in 1 year period due to workers’ health problems. Injury was the highest cause of sick leave days, (2951) (17%), followed by respiratory diseases (2327) (14%). The number of workdays absence by departments was highest among textile department workers, (9027) (53%) followed by garment workers (6415) (38%) and support workers (1481) (9%). In line with this, the proportion of workdays’ absence per number of workers was 2.8, 1.8 and 1.4 in the textile, garment and support respectively.

Disease prevalence across the departments was varied. The prevalence of diagnosis at least for one disease was 69, 65 and 60% among the textile, garment and support department workers respectively. The proportion of textile department workers accounted for 44% of the total workforce; however, the textile department workers’ overall proportion of disease diagnoses is about 49%.

In terms of disease type, a higher percent of respiratory, MSDs, injuries, peptic ulcers, AFI, mouth diseases, skin diseases, eye diseases, allergy, hemorrhoids, and hypertension were identified among the textile department workers (Fig. 2). Gastrointestinal infections, neurological and psychiatric, urinary tract infection, reproductive illnesses and anemia were the highest reported by garment department workers. Respiratory disease was the most prevalent across the three working departments, with 37, 32 and 31% among textile, support and garment departments, respectively (Fig. 2). From the total workers who had at least one respiratory disease, 626 (23%), 135(5%), 84 (3%) and 27 (1%) were diagnosed with bronchitis, asthma, pneumonia and tuberculosis, respectively. MSD was the second most prevalent disorder in all three departments, 31, 28, and 25% among textile, garment, and support respectively. Injury was the third most prevalent issue among the textile department workers, but is the eighth among the garment department workers (Fig. 2).

### Factors associated with the diagnostic category

The statistical analysis uses diseases that were diagnosed at least once for individual workers as a unit of analysis. In the analysis support department, male, education status higher than “read and write” and factory 3 was the reference category. The multivariate logistic regression analysis found that working department (Textile and Garment), sex (female worker), educational status (read and write), age (older worker) and factory type (Factory 1&2) are associated with a higher prevalence of diseases compared to the reference category. Textile department workers have significantly higher odds for eight disease groups with adjusted odds ratio ranges (AOR: 1.22–1.79) compared to the support department workers (Table 3). The garment department workers had significantly higher odds for five disease groups (AOR: 1.30–1.67) compared to the support department workers (Table 3). Female workers had significantly higher odds for seven disease groups (AOR: 1.11–14.76) compared to male workers.

Furthermore, workers with low educational status had significantly higher odds for four disease groups (AOR: 1.36–1.52) than workers of higher education level (Table 3). Age of workers was significantly associated with 14 of the disease groups. Furthermore, factory 1 had significantly higher odds for 14 of the disease groups (AOR: 1.31–42.84), and Factory 2 had significantly higher odds for 11 of the disease groups (AOR: 1.39–38.29) than factory 3 (Table 3).

## Discussion

This study shows that workers in the integrated textile factories were diagnosed with a wide range of diseases in 1 year. Respiratory disease was the leading cause of morbidity followed by MSDs, whereas workplace injuries caused the most days away from work. Working in the textile departments, being female, older age and low educational status are associated with higher risk for most disease groups.

### The size of the problem

The overall disease prevalence in 1 year time is 66%. The majority of the workers were diagnosed at least for one disease in the study period but, some workers had more than one disease diagnosis, which made the total number of consultations for diagnosis 27,320. These figures are higher than reports from other countries. For instance, a cross-sectional study that evaluates the health conditions using clinical examinations of 514 male Indian textile workers found 754 disease conditions, or 1.5 per worker [14]. Another retrospective study from medical records of 1906 workers from mobile clinics in Bangladesh textile and garment reported that 25% of the workers were diagnosed with at least one disease condition [15]. A short survey that examined the occupational health conditions of 845 Indian textile workers found that 447 workers suffered from different diseases [16]. There are several limitations in this comparison with the above studies: difference in the observation period, the difference in diagnosis standard and difference in the study population.

Moreover, in the present study, the proportion of total diagnosed cases from the number of all workers in the factories is 3.4, higher than the proportion of the total number of cases diagnosed from the general population 0.50 in Ethiopia [25]. The total number of cases diagnosed in the general population excluding children less than 5 years of age was 48.8 million, given that the general population’s count of Ethiopia 98.6 million [25]. According to the Ministry of Health annual morbidity statistics report, the annual rate of outpatient visits for a new and repeated health condition is 0.9, which is about four times less than our study population [25]. The result may indicate that workers from the integrated textile factories were diagnosed with more diseases than the general population; however, the high prevalence rate of diagnosis might also be related to free access to health services in factories and other demographic-related differences.

### Workplace factors

Workers in the textile department had a higher prevalence than other employees for many diseases, primarily respiratory diseases. Several other studies from low and middle-income countries have also shown a high prevalence of respiratory problems among textile department workers [7, 16]. An exposure assessment study by Yifokire and colleagues [19] has measured dust exposure levels among the textile production workers and the garment department workers in Ethiopia to be higher than the recommended threshold limit value in the ACGIH [26]. This association might be due to the relationship between respiratory diagnoses and high dust levels in the integrated factories. However, the present study cannot answer this question due to the mixture of diagnoses in the categories used and the lack of exposure measurements in these particular factories. In this study, multiple respiratory diseases were described as bronchitis and asthma; these diagnoses might link with dust exposure.

MSD diagnoses are the second prevalent disease group in the present study and are significantly associated with the manufacturing departments. Both textile and garment department workers have higher odds of MSDs compared to the support department workers. Other studies have highlighted ergonomic hazards in the textile and garment department that could increase the risk of MSDs [27–29]. Additionally, ergonomic hazard exposure assessment studies in Bangladesh and Cambodia found that the tasks in garment production gave a high risk of MSDs [10, 12]. The MSDs may be linked to exposure to ergonomic hazards in the textile and garment departments.

Furthermore, workplace injuries are among the most reported health problems and the primary cause of absence in this study. Both textile and garment department workers handle heavy machinery and have a higher risk of injuries than workers in the support department. This implies that the injuries might be related to the working conditions in the textile and garment departments. However, the prevalence of injuries in this study is lower than in a study of self-reported injuries in another Ethiopian textile factory [20]. The difference may be associated with several factors; one potential reason being that minor injuries managed by first aid may not be included in the diagnostic reports of the factories’ health services.

Moreover, some literature indicates that textile factories have high noise levels in their production departments [9, 18]; consequently, one can expect workers in this department to have a high prevalence of ear problems, as the prolonged noise may cause reduced hearing. However, our study did not show any difference among the textile and support workers regarding ear diseases. However, the diagnosis might not detect reduced hearing among workers in this study, as health offices did not have the equipment to measure hearing ability in the targeted factories. Workers who develop hearing problems may move from the textile department to the support department to reduce their noise exposure or leave the job. Several studies indicated the possibility of self-selection of workers for the job or migration to different departments to mitigate disease [6, 30–32]. Future studies should consider exposure intensity and interruption by tracing the detail of worker’s exposure profiles.

### Personal factors

Sex is significantly associated with most diseases registered in this study; female workers were diagnosed with disease at a higher rate than males. A qualitative in-depth interviewing and focus group discussion with 24 female workers from Bangladesh indicated that female workers suffered from several types of diseases in garment factories [33]. The morbidity assessment study by Singh and colleagues [16] also revealed that female workers in the textile section had more severe anemia than males, similar to the finding of the present study. This might be related to the monthly menstrual cycle among females. Furthermore, a study in Bangladesh has reported a higher prevalence of different diseases among female workers than male workers, but with lower prevalence of injuries [15]. Similarly, the current study shows a lower prevalence of injuries among females; possibly due to differences in tasks, with men often delegated to working with machinery, expose them to a higher risk of injury [20]. Increased morbidity due to MSDs and respiratory diseases were also reported among female textile workers in India [34, 35]. Likewise, a result from the current study found that females are at higher risk of MSDs than males, but found no difference in the likelihood of respiratory diseases.

Previous studies indicated that high disease prevalence among female textile and garment workers could be linked to poor living conditions and engagement in an unhygienic work environment [15, 16, 36–38]. These factors need further study to explore the contexts of this working population.

This study shows that the low educational status of workers in the textile and garment factories is associated with several disease groups, including injuries, MSD, peptic ulcer, UTI, AFI and hemorrhoids. Several studies have also revealed that textile and garment production workers with low educational status are at increased risk of different diseases [16, 38–40]. Another study in India also indicates high overall morbidity among textile and garment workers, which was significantly associated with low educational status [16]. Also, a systematic review indicated that lower educational status could increase the health vulnerabilities of workers [13]. A large study from WHO (*n* = 30,146) also showed that adults’ low educational status was significantly associated with MSD in the LMICs [41]. The increased risk of disease might be associated with the fact that most workers who are low educated are engaged in blue-collar jobs and may not be aware of the presence of different workplace hazards and may have poor access to the safety information at work.

Workers age is also associated with diseases in this study; increased age posing a significantly higher risk for several diseases, including respiratory, MSD, injury, ear diseases and gastrointestinal diseases in comparison to younger workers. Similarly, a study of general health problems among female garment workers in India showed that older age workers have a significantly higher risk for various diseases such as respiratory diseases, gastrointestinal diseases, MSD and eye diseases compared to younger workers [17]. Older workers might be exposed to workplace hazards for many years and have high cumulative exposures. Moreover, workers with work services greater than 5 years had a significantly higher risk for 13 disease groups in the textile department than workers with service less than 5 years. Older factories (1 and 2) had also significantly higher AOR for several disease groups than the recently established factory (3). Therefore, factory clinics can be a good source of information for work-related disease research and action in LMICs setting.

### Different diagnoses than in the general population

The most prevalent cause of morbidity in this study is respiratory health problem (34%), followed by MSD (29%), GI (21%), peptic ulcer (19%) and injuries (17%). The magnitude and type of morbidities are higher and contrast from the general population in Ethiopia. The prevalence of the top leading diseases in the general population of Ethiopia are pneumonia (2.6%), acute upper respiratory infection (2.4%), typhoid (1.7%), dyspepsia (1.6%) and functional intestinal disorder (1.4%) [25]. Unlike the general population, most diseases from the textile and garment department in this study are non-communicable diseases related to dust exposure, ergonomic hazards, contact with chemicals and dangerous machines.

According to the ILO report [23], some of the diseases diagnosed among the textile and garment workers in the integrated factories could be work-related. The diseases are higher in magnitude and different from the diseases found in the general population, especially respiratory diseases, MSD, injury and ear diseases. These diseases might be due to the presence of hazards at the workplaces known to cause those health problems.

According to the “healthy workers effect” concept, a lower morbidity rate is expected among workers compared to the general population. However, the comparison of the morbidity rate of the current study with the general population should be taken cautiously. Generally, workers in integrated factories were distinct from the general population in many ways; they have free access to health service and information, and have a higher average age of 40 years, whereas the average age in the general population is 20 years. In addition, 92% of the study population attended formal education, with only 67% of the general population [42]. This could make a difference in health-seeking behaviour.

In the early nineteenth century, textile workers in the US and Europe suffered from multiple diseases, including a high prevalence of respiratory health problems related to textile cotton exposure [43, 44]. The occupational health and safety standard improvements in developed countries have reduced workers’ health problems [32]. Economic globalization pushed textile factories to developing countries, specifically Asia and Africa. It seems that work-related health problems were exported together with the factories but the improved occupational health and safety standards have been left behind [45].

One of the strengths of this study was the inclusion of workers in the factories from all three work departments of textile, garment, and support. However, we do not know how representative the disease figures are regarding actual prevalence since the workers may also visit other health institutions. Conversely, the factory clinics serve workers free of charge and have a referral to hospitals for advanced diagnosis and treatment; thus, it is very likely that workers can consult the factory clinics to a large extent.

On the other hand, the main weakness of this study was the lack of standard diagnostic codes in the archives from the health clinics that forced us to use large categories for diagnoses. We also had limited information about the worker’s exposure profile and could not collect potential confounder variables such as previous health condition, current work exposure at the different departments, housing, living environment, family, behaviour and lifestyle-related information. It is subsequently difficult to know the root causes of various health problems. Using a control group from another industry might have improved the study. Comparing groups inside the factory have advantages to link health problems to work conditions as the workers in the three departments had the same organizational experiences and the same factory culture. There still however could be self-selection of workers for the job, and movement of workers within the departments attributed to health conditions could be an inherent problem.

## Conclusion

About two-thirds of the workers in the integrated textile factories are diagnosed with different types of diseases, with a high prevalence of morbidity. The textile and garment department workers suffered a higher prevalence of diseases than support department workers, indicating that some diseases might be related to work in these departments. Work department, sex, age and educational status were significantly associated with several registered work-related diseases. Besides, respiratory, MSD, injuries and ear diseases were higher in quantity than the general population. Factory clinics seem to be an essential source of evidence to understand the occupational disease burden. Comparison of the risk level among the working departments and the general population needs careful attention while interpreting the result due to the lack of control for the potential confounders. Further study is needed to investigate the reason for repeated clinic consultation and rare chronic diseases such as cancer, heart diseases, renal diseases, and diabetics in relation to worker’s exposure profile.

## Data Availability

The datasets generated and analyzed during the current study are not publicly available because the information may be linked to the business firms but are available from the corresponding author on reasonable request.

## Abbreviations

ACIGH: American Conference of Government Industrial Hygienists
AFI: Acute febrile illness
AORs: Adjusted odds ratios
CIs: Confidence intervals
ILO: International Labor Organization
ITF: Integrated textile factory
LMICs: Low and middle income countries
MSDs: Musculoskeletal disorders
NORHED: Norwegian Program for Capacity Building in Higher Education and Research for Development
OHS: Occupational health service
COR: Crude odds ratio
SPSS: Statistical Package for Social Sciences
USA: United States of America
UTI: Urinary tract infection
WHO: World Health Organization
